# Age-dependent neurological phenotypes in a mouse model of PRRT2-related diseases

**DOI:** 10.1007/s10048-021-00645-6

**Published:** 2021-06-08

**Authors:** Fay AJ, McMahon T, Im C, Bair-Marshall C, Niesner KJ, Li H, Nelson A, Voglmaier SM, Fu Y-H, Ptáček LJ

**Affiliations:** 1 Department of Neurology, University of California San Francisco, San Francisco, CA 94143 USA; 2 Department of Psychiatry and Behavioral Sciences, University of California San Francisco, San Francisco, CA 94143 USA; 3 Weill Institute for Neuroscience, University of California San Francisco, San Francisco, CA 94143 USA; 4 Kavli Institute for Fundamental Neuroscience, University of California San Francisco, 548F Rock Hall, MC-2922, 1550 4th Street, San Francisco, CA 94143 USA; 5 Institute for Human Genetics, University of California San Francisco, San Francisco, CA 94143 USA

**Keywords:** Dyskinesia, Movement disorders, Epilepsy, Mouse model, Paroxysmal disorders

## Abstract

Paroxysmal kinesigenic dyskinesia is an episodic movement disorder caused by dominant mutations in the proline-rich transmembrane protein PRRT2, with onset in childhood and typically with improvement or resolution by middle age. Mutations in the same gene may also cause benign infantile seizures, which begin in the first year of life and typically remit by the age of 2 years. Many details of PRRT2 function at the synapse, and the effects of mutations on neuronal excitability in the pathophysiology of epilepsy and dyskinesia, have emerged through the work of several groups over the last decade. However, the age dependence of the phenotypes has not been explored in detail in transgenic models. Here, we report our findings in heterozygous and homozygous Prrt2 knockout mice that recapitulate the age dependence of dyskinesia seen in the human disease. We show that Prrt2 deletion reduces the levels of synaptic proteins in a dose-dependent manner that is most pronounced at postnatal day 5 (P5), attenuates at P60, and disappears by P180. In a test for foot slippage while crossing a balance beam, transient loss of coordination was most pronounced at P60 and less prominent at age extremes. Slower traverse time was noted in homozygous knockout mice only, consistent with the ataxia seen in rare individuals with biallelic loss of function mutations in Prrt2. We thus identify three age-dependent phenotypic windows in the mouse model, which recapitulate the pattern seen in humans with PRRT2-related diseases.

## Introduction

Mutations in the proline-rich transmembrane protein PRRT2 were implicated in several paroxysmal neurological disorders almost a decade ago,[1–5] and over the past several years, a more detailed understanding of the functions of this protein has emerged. PRRT2-related disorders include dominant paroxysmal kinesigenic dyskinesia [6] and benign familial infantile seizures, with rare manifestations of episodic ataxia and hemiplegic migraine. [7, 8] In addition, a small number of patients have been identified who carry biallelic mutations in PRRT2, resulting in a more severe phenotype that includes intellectual disability, developmental delay, ataxia, and paroxysmal dyskinesias.[9] PRRT2 mutations were found primarily to lead to a loss of function through nonsense-mediated decay, pointing to a haploinsufficiency mechanism. [5] This is further supported by the more severe phenotype in individuals with homozygous mutations. [7] The PRRT2 protein localizes to the presynaptic membrane, where it interacts with components of the calcium-dependent vesicle release machinery, including the SNARE complex proteins VAMP2, syntaxin, and synaptotagmin-1 and synaptotagmin-2. [10] PRRT2 exerts an inhibitory effect on vesicle release at synapses, particularly glutamatergic synapses, [11] and possibly alters release of other neurotransmitters, including GABA. [12] A more recent study suggests that PRRT2 may also serve an important function in regulating voltage-gated sodium channels at the initial segment of glutamatergic neuronal axons, such that partial loss of PRRT2 leads to excessive sodium channel activity and hyperexcitability of these neurons. [13].

A unique and poorly understood feature of PRRT2-related diseases is the age dependence of the various phenotypes: seizures tend to occur in the infantile period [14] and abate within the first 2 years, whereas paroxysmal kinesigenic dyskinesia typically appears among school-age children, and may continue through mid-adulthood, after which time it often improves. [15] Knockout mice [16–18] made by several groups have recapitulated features similar to the human phenotypes, but prior studies have explored neither the age dependence of the phenotypes nor the mechanisms for the age dependence of seizures and dyskinesia. In this study, we explore the effects of PRRT2 heterozygous and knockout states in mice, including the age-dependent expression of synaptic proteins and motor manifestations.

## Experimental methods

### Nomenclature

In this study, standard nomenclature for names of genes and proteins was used. *PRRT2* represents the human gene name, while PRRT2 is the human protein name and PKD the acronym for the disorder (paroxysmal kinesigenic dyskinesia). *Prrt2* is the mouse gene name, and Prrt2 is the name for the mouse protein. “Pkd mice” is used to denote the animal model we created that harbored the PKD phenotype with a heterozygous loss of expression in one allele of the mouse *Prrt2*. “Prrt2 KO mice” is used to denote that the animal model has a homozygous loss of expression in both alleles of the mouse *Prrt2.*

### Generation of paroxysmal kinesigenic dyskinesia model mice

All studies were performed following protocols approved by the University of California San Francisco Institutional Animal Care and Use Committee. The Prrt2 knockout-first KOMP ES-cell line was purchased, expanded, genotyped, and karyotyped by UC Davis KOMP Repository. The *Prrt2*^*tm1a(KOMP)Wtsi*^ chimera mice in a C57BL/6 J background were generated by blastocyst injection of targeted ES-cell clone JM8A3.N1 subline at the Gladstone Institute Transgenic Gene Targeting Core. The *Prrt2* knockout-first, KOMP allele (reporter-tagged insertion with conditional potential) uses a construct that introduces a floxed locus so that cell type–specific KO can be achieved through breeding with Cre recombinase mice. The knockout-first allele is flexible and can produce reporter knockouts, conditional knockouts, and null alleles following exposure to site-specific recombinases Cre and Flp. The “mouse *En2* splice acceptor and the SV40 polyadenylation sequences are highly efficient in producing a null allele without cre or flp” (ftp://ftp.sanger.ac.uk/pub/resources/opendoor/washington/2011/manuals/module4_ikmc.pdf).

The chimeras were bred to C57BL/6 J mice to obtain germline transmission and maintained in our animal facility at The University of California San Francisco. For this study, we used the heterozygous and homozygous knockout-first mouse line. *Prrt*2 KO-first mice were genotyped by tail qPCR using primers designed to the allele-specific *Prrt2* gene trap, neo, lacz, and sex.

PCR genotyping of mouse tail DNA was performed with the following primers:

Prrt2.5wt_F: 5′-AAG GGT ACA GAA GGA GCA GAC TG-3′,

Prrt2.5wt_R: 5″-CCC AAC AAG ACC CAG TCT CTC-3′,

Prrt2.gt_F: 5′-CTG ACG CAT GCA CAG ATT TTC-3′,

Prrt2.gt_R: 5′-GGA ACT TCG GAA TAG GAA CTT CG-3′.

PCR products were 129 and 139 bp for the wild-type and gene-trapped (knockout-first) allele, respectively. All samples were run with a 5 uL reaction volume containing 2 X EXPRESS SYBR GreenER qPCR SuperMix, primers at final concentration of 0.3 uM each, water to a 4.8 uL final volume, and 0.2 uL of genomic DNA. The PCR was run in the QuantStudio™ 6 Flex Real-Time PCR System (Applied Biosystems) using the following amplification parameters: 2 min at 95 °C, and 35 cycles of 15 s at 95 °C and 30 s at 60 °C, then followed by 15 s at 95 °C, 1 min at 60 °C, and 15 s at 99 °C. The genotype was analyzed by Ct (cycle threshold) values and dissociation/melt curves after PCR cycles.

Each sample was also checked for sex using X and Y chromosome-specific primers; Htr2c-3_WT_F: 5′-TAC TAC CTG CGT GCT CAA TGA C-3′, Htr2c-3_WT_R: 5′-CAG GCT GAT ATT ACG CAG TTC C-3, Sry_F_tm: 5′-AAT GCC ACT CCT CTG TGA CAC T-3′, and Sry_R_tm: 5′-CAG GAG GCA CAG AGA TTG AAG A-3′. PCR reaction mix and cycles are the same as above.

#### Balance beam

Experimenters were blinded to genotype until the behavioral assays were completed. Mice were placed at the end of an 18, 12, or 6-mm beam and trained to run into a dark box, across the beam, for a total period of 8 days. The height of the beam (50 cm above the ground) and a bright light at the end of the beam served as aversive stimuli. The mice were set to cross from the thickest beam to the thinnest with at least a 15-min break period in between. Mice were trained for the first 4 days. On testing days (5–8), the time to traverse each beam was recorded. Fifteen-minute intertrial breaks were given. If a mouse took longer than 60 s to cross or fell off the beam, it was scored as 60 s. Trials in which mice stopped or turned around were repeated. The traverse time and number of slips were recorded. Naïve mice were tested at each time point (postnatal day 27–30, 60, and 180).

#### Rotarod

Experimenters were blinded to genotype until the behavioral assays were completed. Each mouse was gently transferred to the rotarod as it rotated at 4 RPM and allowed to acclimate for several seconds. The apparatus was set to accelerate from 4 to 40 RPM over 300 s. The apparatus was switched to accelerate as the timers were reset. The time to fall, with a maximum of 300 s, was recorded once daily for 8 days.

#### Gait analysis

To obtain footprints, the hind-feet and fore-feet of the mice were coated with red and black nontoxic paints, respectively. The animals were then allowed to walk along a 100-cm long, 10-cm wide runway (with 10-cm-high walls) into an enclosed box, and a sheet of millimeter paper was placed on the floor of the runway. To characterize the walking pattern of each mouse, the average distance between each stride (stride length, SL), the distance between left and right hind footprints (hind-base width, HB), and the distance between left and right fore footprints (fore-base width, FB) were measured. [19].

#### Western blot

Mouse cerebellum tissue was collected from mice at various ages and snap frozen on liquid nitrogen and subsequently stored at − 80 °C. Total cellular proteins were extracted using RIPA buffer (150 mM NaCl, 1% NP-40, 0.1% SDS, 0.5% sodium deoxycholate, 20 mM Tris, pH 7.5, and 5 mM EDTA) containing cOmplete protease inhibitors (Sigma-Aldrich). Protein lysates from cells were prepared in SDS-PAGE loading buffer. Equal amounts of protein were resolved on 4–12% SDS-PAGE gels and then transferred to nitrocellulose membrane. After blockage and incubation with primary antibody in 2% BSA: TBST at 4 °C overnight, membranes were incubated with secondary antibodies at room temperature for 1 h. The primary antibodies were anti-vGlut1 mouse monoclonal antibody (1:500; Novus; Littleton, CO), anti-VAMP2 mouse monoclonal antibody (1:1000; Novus; Littleton, CO), anti-NSF rabbit polyclonal antibody (1:1000; Cell Signaling; Danvers, MA), anti-SNAP25 rabbit polyclonal antibody (1:1000; Cell Signaling), anti-Synaptotagmin mouse monoclonal antibody (1:1000; Abcam; Cambridge, MA), anti-Complexin 1/2 rabbit polyclonal antibody (1:1000; Cell Signaling; Danvers, MA), anti-MUNC18-1 mouse monoclonal antibody (1:1000; SYSY; Göttingen, Germany), anti-Syntaxin 1 mouse monoclonal antibody (1:1000; SYSY), and anti-Syntaxin 2 antibody (1:1000; Abcam). The Prrt2 antibody was generated by immunizing rabbits using a synthesized oligopeptide (TQSDPQPTSQPPPKPPLQA) (Biomatik; Wilmington, DE), corresponding to a 19 amino acid central portion of the mouse Prrt2 gene (1:2000; Agbio, Inc; Lodi, CA). All blots were normalized using anti-GAPDH mouse monoclonal antibody (1:20,000; Life Technology). Membranes were incubated with secondary antibodies (conjugated either with IRDye 680 or with IRDye 800, LI-COR Biosciences; Lincoln, NE) at room temperature for 1 h and visualized with an Odyssey Infrared Imaging System (LI-COR Biosciences). Experiments were run at least three times. Results were expressed as a percentage of its wild-type (WT) mean values.

#### rt-qPCR

At the appropriate age, mice were anesthetized with Avertin (600 mg/kg), and their brains were removed and immediately dissected on ice before preservation in RNALater. The brain stem with midbrain, cerebellum, anterior half of the cortex, hippocampus, spinal cord, striatum, and thalamus were separated and individually analyzed for both wild-type and hemizygous mice. Samples were removed from RNALater 1–2 days later and stored at − 80 °C. mRNA was isolated (Qiagen RNEasy Lipid Mini Kit) and converted to cDNA (Qiagen SuperScript IV). qPCR was done using probes for Prrt2 exon 2–3 (Mm.PT.58.7964149.g, IDT) and normalized to GAPDH (Mm.PT.39a.1**,** IDT).

#### Primary cerebellar granule cell cultures

Thirty-five-millimeter tissue culture plates were coated with poly-l-ornithine (0.1 mg/mL, overnight) and gelatin (250 µg/mL, overnight) before plating neurons. Mouse cerebellar granule cells were isolated at P5 using a Papain Dissociation System (Worthington Biochemical Corporation) and plated at 1 million cells per 35-mm plate in serum-free media (SFM): Neurobasal A medium (Invitrogen, Carlsbad, CA), 1 × GlutaMAX I (Fisher Scientific), 2% B27 serum-free supplement, 250 µM KCL, penicillin (100 U/mL) (Invitrogen, Carlsbad, CA), and streptomycin (100 ug/mL) (Invitrogen, Carlsbad, CA). After 3–5 days in vitro (DIV), incubated in a humidified 5% CO_2_⁄95% air atmosphere at 37 °C, half the medium was removed and cells were treated with cytosine arabinoside (8 µM) in SFM to inhibit non-neuronal cell proliferation. Subsequently, half of the medium was changed every third day. Cells were used for ELISA analysis on day 14, at which time-cultured cerebellar granule cells express functional glutamate receptors. [20–23] Residual GABA release is likely due to low levels of contamination of the culture with Purkinje cells and interneurons.

#### ELISA analysis

Cerebellar neurons were processed for measurements of glutamate concentration and GABA concentration following the manufacturer’s protocols for glutamate ELISA kit (Cell Biolabs Inc, San Diego, CA) and the GABA ELISA kit (Cloud-Clone Corp, Houston, TX), respectively. In short, the P5 cerebellar neuron cultures were removed 14 days after incubation, and the cultures were washed once in loading buffer (LB) (150 mM NaCl, 2 mM KCL, 1.2 mM CaCL2, 1 mM MgCL2, 1 mM NaHPO4, 25 mM Glucose, and 10 mM HEPES, pH 7.3) warmed to 37 °C. The LB was replaced with 0.5 mL of 37 °C LB and allowed to equilibrate for 60 (glutamate release) or 120 (GABA release) min. After the incubation period, samples were collected and centrifuged and placed on ice. Plates were scraped in ice-cold LB with protease inhibitors, briefly sonicated and centrifuged to rid of cellular debris. Samples were taken for Bradford analysis of protein levels and ELISA to ascertain part of the total neurotransmitter levels. Results were adjusted for protein levels and expressed as a percentage of total neurotransmitter level (neurotransmitter release/neurotransmitter release + lysate level × 100).

### Exocytosis analysis

#### Reagents:

Bafilomycin 1A was obtained from Calbiochem (EMD Millipore, San Diego, CA, USA). CPP (3-(2-carboxypiperazin-4-yl) propyl-1-phosphonic acid) and CNQX (6-cyano-7 nitroquinoxaline-2,3-dione) were purchased from Tocris Bioscience (Ellisville, MO).

#### Primary mouse hippocampal culture and transfection*:*

Hippocampi from P0 pups of either sex were dissected and dissociated after confirmation of genotype by PCR from tail DNA. Hippocampal tissues were digested using 200 U papain (Worthington) in HBSS buffer containing 0.2 mg/mL l-cysteine (Fluka), 500 μM EDTA, 1 mM CaCl_2_, and 3 mM NaOH. Digestion was terminated with 2.5 mg/mL soybean trypsin inhibitor (Worthington), and 2.5 mg/mL bovine serum albumin (BSA) in minimum essential media (MEM; ThermoFisher) growth solution. MEM growth solution consists of MEM with 5% fetal bovine serum (FBS), 2% B-27, 1% GlutaMax (Invitrogen), 0.1% MITO + Serum Extender (BD Biosciences), and 21 mM glucose (Sigma-Aldrich). Mouse neurons were transfected with VGLUT1-pHluorin in a pCAGGs vector (Voglmaier Lab), using the Basic Neuron SCN Nucleofector kit, according to the manufacturer’s directions (Lonza, Walkersville, MD). Cells were maintained in Neurobasal media supplemented with 1% heat inactivated FBS, 10% NeuroMix growth supplement (PAA, Dartmouth, MA), 2 mM GlutaMax, 15 mM NaCl, and 10 μg/mL primocin antibiotic (Lonza) and imaged at 14–16 days in vitro (DIV). 5-Fluoro-2′-deoxyuridine (10 μM final concentration) was added at DIV5–7 as a mitotic inhibitor to control glial growth.

#### Live cell imaging:

Live cell imaging was performed essentially as described previously. [24] Coverslips with transfected hippocampal neurons were mounted in a rapid-switching, laminar-flow perfusion and stimulation chamber (Warner Instruments, Holliston, MA) on an inverted epifluorescence microscope (Nikon, Melville, NY) and imaged at room temperature using a 63X oil objective (NA = 1.4). Cells were imaged in modified Tyrode’s solution pH 7.4 (in mM: 119 NaCl, 10 HEPES–NaOH, 30 glucose, 2.5 KCl, 2 CaCl_2_, 2 MgCl_2_) containing 10 μM each of the glutamate receptor inhibitors CNQX and CPP. Electrical stimulation to elicit action potentials [25] was applied using an A310 Accupulser (WPI, Sarasota, FL) at 5–100 Hz with 1-ms bipolar current pulses through platinum-iridium electrodes, to yield fields of 5–10 V/cm across the chamber. [22] Cells were illuminated using a Xenon lamp (Sutter Instruments, Novato, CA) with a 470/40-nm excitation and a 525/50-nm emission filter (Chroma, Bellows Falls, VT). Images were acquired on a QuantEM CCD camera (Photometrics, Tuscon, AZ), exposing the fluorophore for 300 ms, and images were collected every 3 s. The MetaMorph software was used to control data collection and to perform offline analysis (Universal Imaging, Sunnyvale, CA). The total pool size was determined using Tyrode’s solution with 50 mM NH_4_Cl (NaCl reduced by 50 mM to compensate). To measure exocytosis alone, cultures were incubated in modified Tyrode’s medium containing 0.5–1 µM bafilomycin A for 30 s before imaging in the same medium.

#### Data analysis:

As described previously, [22, 26] the MetaMorph software was used to quantify the average fluorescence of regions of interest (ROI) in 4 × 4 pixel boxes placed over the center of manually selected boutons. The average fluorescence of three 4 × 4 pixel ROIs without cellular elements was subtracted as background. Baseline values from the first 5 frames (before stimulation) were averaged as initial fluorescence *F*_0_, and the dynamics of fluorescence intensity expressed as fractional change (Δ*F*) over initial fluorescence. For normalized measurements, the average pHlourin fluorescence over individual boutons was normalized to the total fluorescence as visualized by application of modified Tyrode’s solution containing 50 mM NH_4_Cl to alkalinize all synaptic compartments. Fluorescence measurements from 27 to 91 boutons per coverslip were averaged, and the means from 7 to 10 coverslips from three independent cultures were averaged. Data are presented as means ± SEM. Significance of differences between groups was assessed by two-tailed, unpaired *t* test at *p* < 0.05 where appropriate (GraphPad Prism). The fraction of transporter that undergoes exocytosis (recycling pool, RP) was measured as the fraction of the total pool that undergoes exocytosis in response to 10-Hz 90-s stimulation. [27] The rate of exocytosis [(Δ*F*/*F*_0_)/s] was estimated from a linear fit to the increase in pHluorin fluorescence during the initial 15 s of stimulation in the presence of bafilomycin.

### Slice electrophysiology

We prepared sagittal sections of the cerebellum from Prrt2 mice aged 1 month (+ / − 3 days), 2 months (+ / − 7 days), or 6 months (+ / − 14 days). Recordings from wild-type, heterozygous, and knockout littermates were performed in an interleaved fashion.

Mice were then deeply anesthetized with an IP ketamine-xylazine injection, transcardially perfused with ice-cold sucrose-based or glycerol-based slicing solution, decapitated, and the brain was removed, mounted on a chuck, and submerged in a slicing chamber with ice-cold slicing solution. Glycerol slicing solution contained (in mM) 250 glycerol, 2.5 KCl, 1.2 NaH_2_PO_4_, 10 HEPES, 21 NaHCO_3_, 5 glucose, 2 MgCl_2_, and 2 CaCl_2_. Sequential 300-μm sagittal slices were cut on a vibrating microtome (Leica), transferred to a chamber of warm (34 °C) carbogenated ACSF containing (in mM) 125 NaCl, 26 NaHCO_3_, 2.5 KCl, 1 MgCl_2_, 2 CaCl_2_, 1.25 NaH_2_PO_4_, and 12.5 glucose for 30–60 min, then stored in carbogenated ACSF at room temperature. Each slice was then submerged in a chamber superfused with carbogenated ACSF at 31–33 °C for recordings.

Purkinje cells were targeted for recordings using differential interference contrast (DIC) imaging. Neurons were patched in the whole-cell voltage-clamp configuration using borosilicate glass electrodes (3–5 MΩ) filled with cesium-based (voltage-clamp) internal solution containing (in mM) 120 CsMeSO_3_, 15 CsCl, 8 NaCl, 0.5 EGTA, 10 HEPES, 2 MgATP, 0.3 NaGTP, 5 QX-314, and pH 7.3. Picrotoxin was added to the external solution to block synaptic currents mediated by GABA_A_ receptors. Tetrodotoxin (TTX) was added to the external solution to block action potential–dependent synaptic release.

Whole-cell recordings were made using a MultiClamp 700B amplifier (Molecular Devices) and ITC-18 A/D board (HEKA). Data was acquired and analyzed using the Igor Pro 6.0 software (Wavemetrics) and custom acquisition/analysis routines (mafPC, courtesy of MA Xu-Friedman). Whole-cell recordings were filtered at 2 kHz and digitized at 10 kHz. Synaptic currents were monitored at a holding potential of − 70 mV. Series resistance and leak currents were monitored continuously. Miniature EPSCs (mEPSCs) were recorded at –70 mV in TTX and picrotoxin. mEPSC events were identified by their current amplitude (> 5 pA) and the first derivative of the current amplitude (mafPC). Only cells with at least 500 mEPSC events were included in the analysis.

Evoked EPSCs onto Purkinje cells were elicited in the presence of picrotoxin with a stimulus isolator (IsoFlex, AMPI) and a glass electrode placed in the nearby molecular layer. Stimulus intensity was adjusted to yield EPSC amplitudes of approximately 400 pA. Stimulus duration was 300 μs. For evaluation of the paired-pulse ratio, two stimuli were given at variable interstimulus intervals (ISIs; 25, 50, 100, 200, 500 ms) with a 20-s intertrial interval. Three repetitions at each ISI were averaged to yield the PPR for that ISI.

## Results

**Fig. 1:**
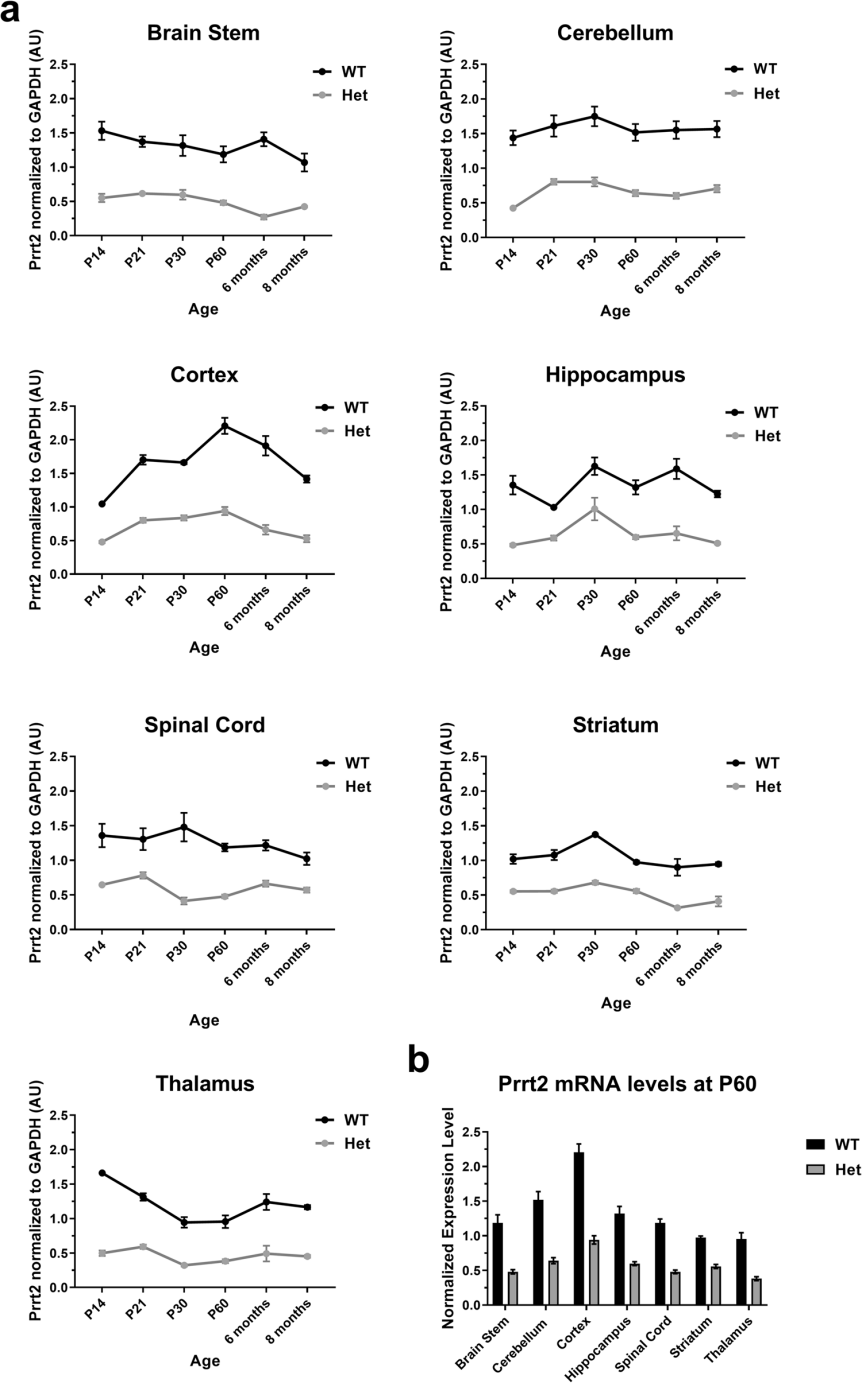
Confirmation of the quantitation and distribution of *Prrt2* mRNA throughout the central nervous system with an allelic mutation–dependent reduction in Pkd and Prrt2 KO mice. Prrt2 mRNA levels were assessed by quantitative real-time reverse transcriptase PCR in several brain regions of mice. **a** From postnatal day 14 to 8 months of age. Prrt2 wild-type mice (WT *n* = 3–5, black line) versus Het mice (*n* = 3–6, grey line). mRNA levels of the Prrt2 het mice were approximately half that of their WT littermates. All samples were normalized to GAPDH controls. **b** Prrt2 mRNA expression levels at postnatal 60 (P60) in several brain regions were highest in the cortex, followed by the cerebellum, with relatively similar expression levels in the thalamus, hippocampus, brainstem, striatum, and spinal cord. All comparisons were significant by genotype by two-way ANOVA ****p < 0.0001.

Figure 1 shows that mRNA levels in the Pkd mice are approximately half of levels in wild-type mice across several brain regions and in mice ranging from postnatal day 14 to 8 months of age. Expression at postnatal day 60 (P60) demonstrates that mRNA levels, normalized to GAPDH mRNA, are highest in the cortex, followed by the cerebellum, with relatively similar expression levels in the thalamus, hippocampus, brainstem, striatum, and spinal cord. Expression reaches a peak around P30 in the cerebellum, striatum, hippocampus, and spinal cord, while in the cortex, expression is maximal around P60. In the cortex, there is a U-shaped expression pattern over time, whereas mRNA levels vary less across age in other parts of the central nervous system.

**Fig. 2:**
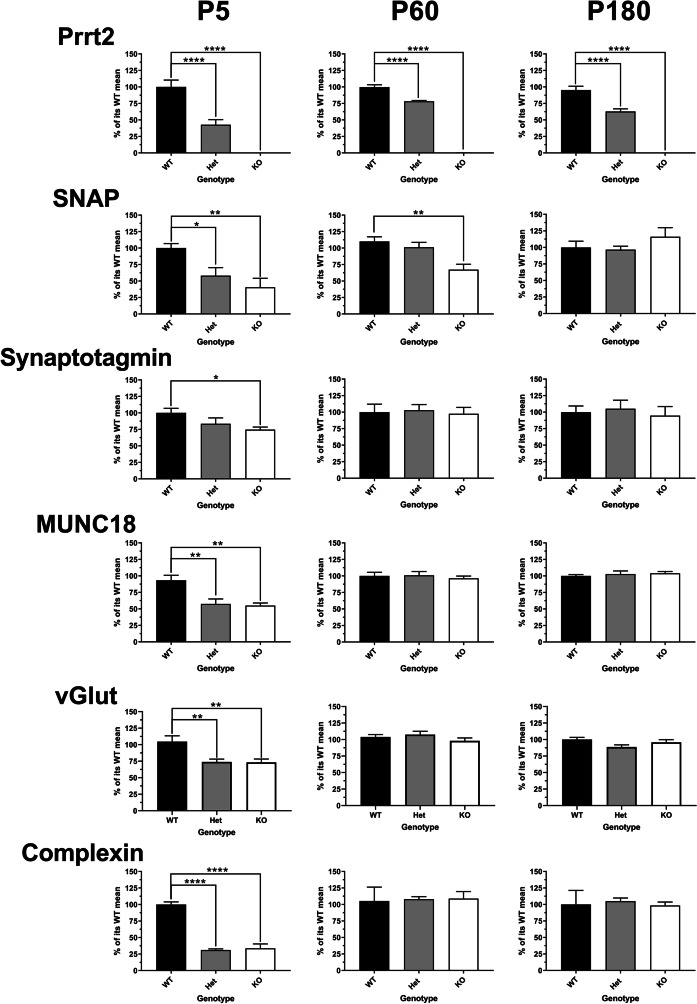

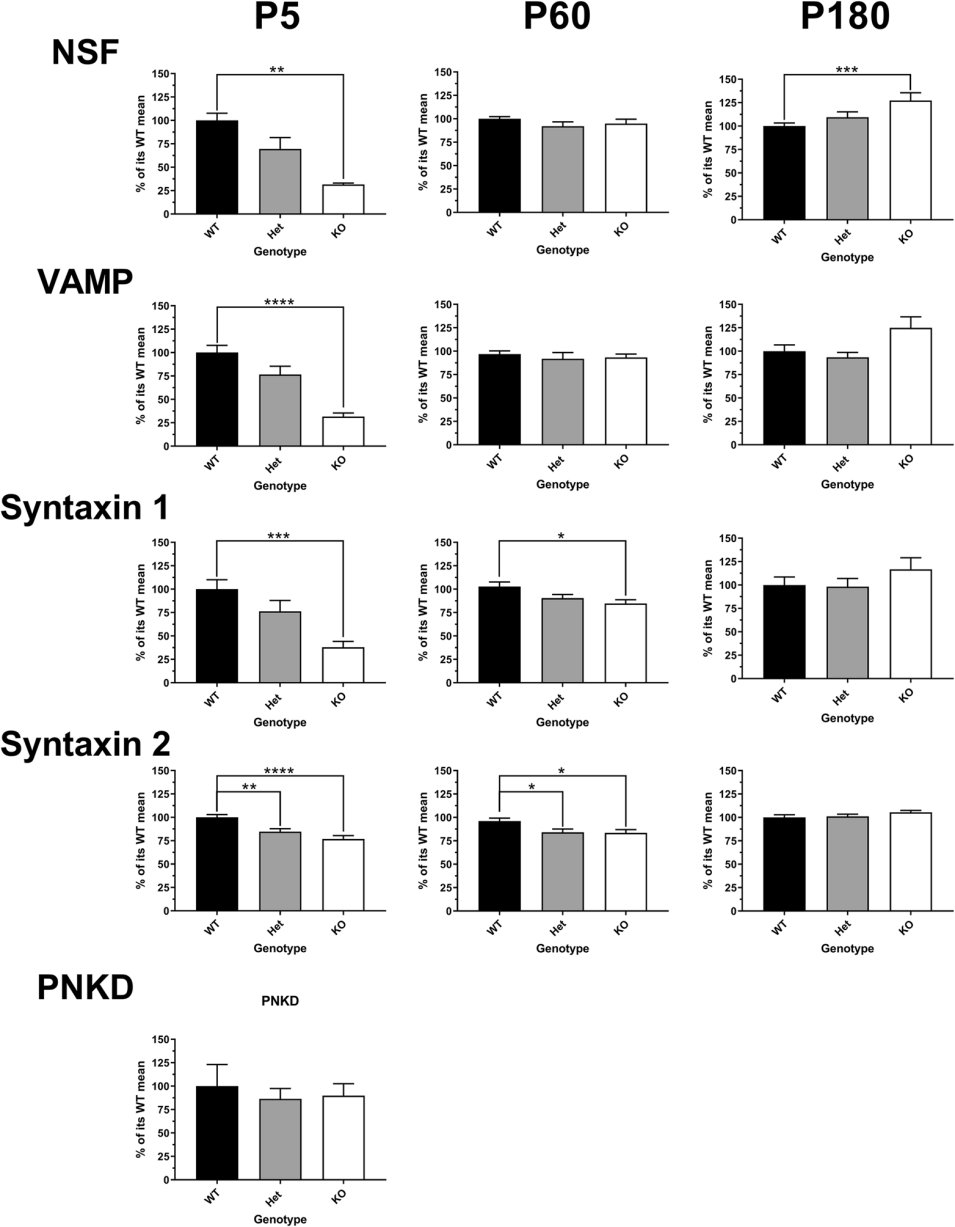
At the earliest time point studied, in P5 cerebellar neurons, *Prrt2* mutation is associated with reductions of proteins associated with the SNARE complex. Followed by small reductions in a few proteins of the SNARE complex at P60 and those reductions are mitigated at P180. Western blot analysis of Prrt2 and SNARE complex proteins of cerebellar tissue in Prrt2 WT (black bars), Het (grey bars), and KO (white bars) mice. Samples were first normalized to GAPDH; then comparisons were made to the mean value of Prrt2 WT mice and expressed as % of its WT mean + / − S.E.M. In postnatal 5 days (P5) old, there was a significant reduction in *Prrt2*: WT *n* = 12, Het *n* = 9, KO *n* = 13; *SNAP25*: WT *n* = 8, Het *n* = 8, KO *n* = 8; *Synaptotagmin*: WT *n* = 17, Het *n* = 18, KO *n* = 18; *MUNC18*: WT *n* = 6, Het *n* = 6, KO *n* = 6; *vGlut1*: WT *n* = 10, Het *n* = 8, KO *n* = 10; *Complexin 1* + *2*: WT *n* = 3, Het *n* = 3, KO *n* = 3; *NSF*: WT *n* = 3, Het *n* = 3, KO *n* = 3; *VAMP*: WT *n* = 12, Het *n* = 12, KO *n* = 12; *syntaxin 1*: WT *n* = 12, Het *n* = 12, *n* = 12; *syntaxin 2*: WT *n* = 12, Het *n* = 12, KO *n* = 12; *PNKD*: WT *n* = 5, Het *n* = 5, KO *n* = 5. In postnatal 60 days (P60) old, there was a significant reduction in *Prrt2*: WT *n* = 6, Het *n* = 6, KO *n* = 6; *SNAP25*: WT *n* = 10, Het *n* = 9, KO *n* = 10; *Synaptotagmin*: WT *n* = 6, Het *n* = 6, KO *n* = 6; *MUNC18*: WT *n* = 6, Het *n* = 6, KO *n* = 6; *vGlut1*: WT *n* = 10, Het *n* = 11, KO *n* = 11; *Complexin 1* + *2*: WT *n* = 9, Het *n* = 8, KO *n* = 9; *NSF*: WT* n* = 6, Het *n* = 6, KO *n* = 6; *VAMP*: WT *n* = 12, Het *n* = 12, KO *n* = 12; *syntaxin 1*: WT *n* = 8, Het *n* = 9, KO *n* = 9; *syntaxin 2*: WT *n* = 9, Het *n* = 9, KO *n* = 9. In postnatal 180 days (P180) old, there was a significant reduction in *Prrt2*: WT *n* = 6, Het *n* = 6, KO *n* = 6; *SNAP25*: WT* n* = 18, Het *n* = 18, KO *n* = 18; *Synaptotagmin*: WT *n* = 6, Het *n* = 6, KO *n* = 6; *MUNC18*: WT *n* = 6, Het *n* = 6, KO *n* = 6; *vGlut1*: WT *n* = 12, Het *n* = 12, KO *n* = 12; *Complexin 1* + *2*: WT* n* = 18, Het *n* = 18, KO *n* = 18; *NSF*: WT *n* = 24, Het *n* = 22, KO *n* = 24; *VAMP*: WT *n* = 12, Het *n* = 12, KO *n* = 12; *syntaxin 1*: WT *n* = 6, Het *n* = 6, KO (*p* = NS, *n* = 6); *syntaxin 2*: WT *n* = 12, Het *n* = 12, and KO *n* = 12. All comparisons were done by one-way ANOVA, Tukey’s multiple comparisons, compared to that in WT. (*p < 0.05, **p < 0.01, ***p < 0.001, ****p < 0.0001)

A previous work has identified PRRT2 localization at the synapse, and co-localization with proteins of the SNARE complex that are essential for neurotransmitter release. [8, 9] We next sought to determine the effect on expression levels of several presynaptic proteins in knockout and heterozygous mice compared to that in wild-type. At the earliest time point studied, in P5 cerebellar neurons, PRRT2 mutation is associated with reductions of SNARE complex components SNAP-25, syntaxin-1, syntaxin-2, synaptobrevin, Munc-18, complexins 1 and 2, and synaptotagmin, as well as vesicle-associated proteins vGLUT-1 and synaptotagmin, and the cytoplasmic ATPase NSF. In Pkd mice, the reductions in some synaptic proteins, including SNAP-25, syntaxin, NSF, and synaptobrevin, appear to be dose-dependent with PRRT2 (Fig. 2a). In other cases, the reduction in protein expression is similar in both the Prrt2 KO and Pkd mice, as is the case for vGLUT1, Munc18, complexins 1 and 2, syntaxin-2, and synaptotagmin. By P60, there are no longer any differences in expression of vGLUT1, complexins 1 and 2, Munc18, NSF, synaptobrevin, or synaptotagmin (Fig. 2b). Small reductions in SNAP-25, syntaxin-1, and synatxin-2 remain at P60. At P180 (Fig. 2c), reductions in protein levels of the above synaptic proteins are no longer detectable in the Pkd and Prrt2 KO compared to that in wild-type. In fact, there are mild increases in the levels of NSF and synaptobrevin in the Prrt2 KO mice versus wild-type at this later time point.

**Fig. 3:**
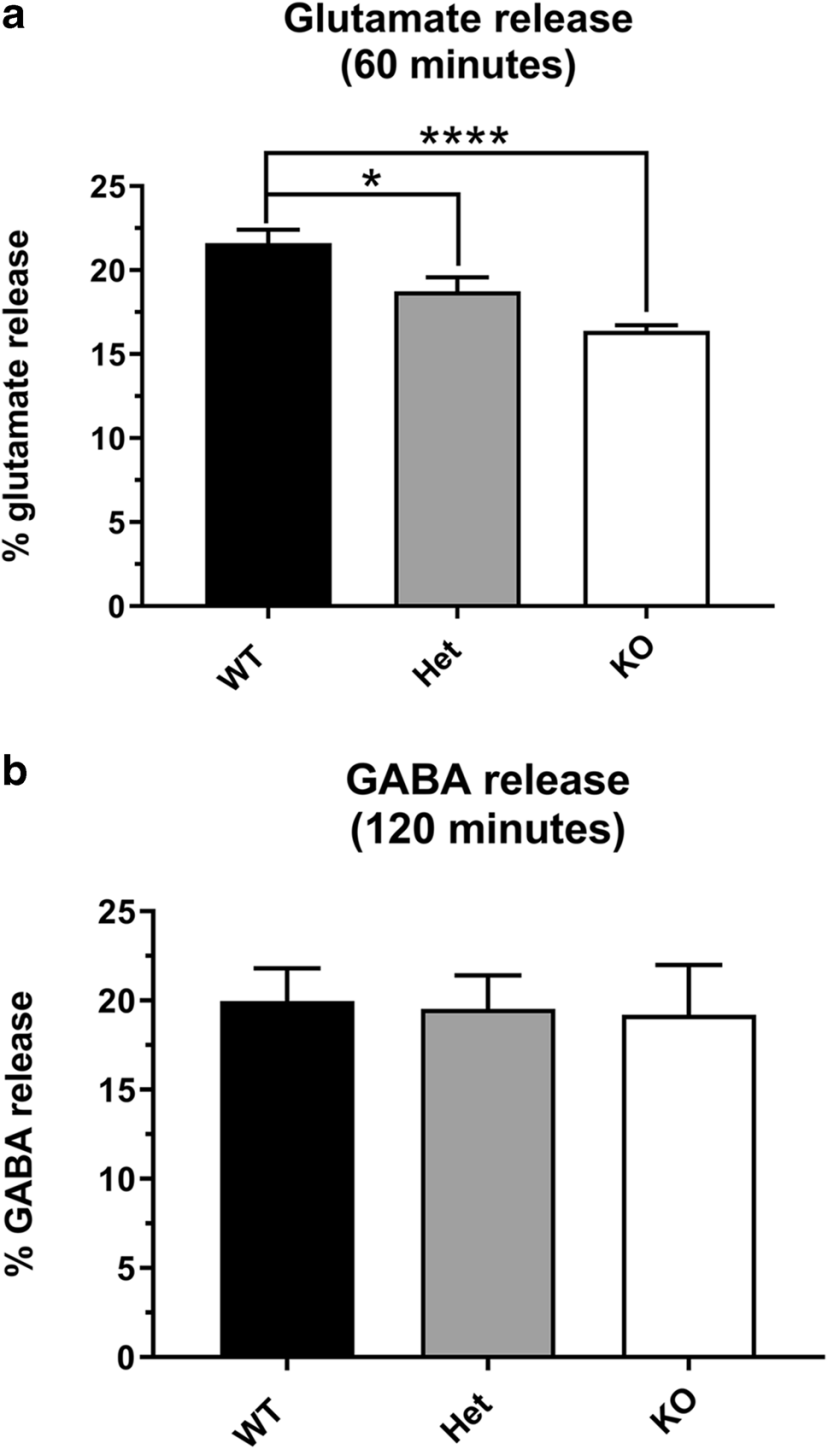
Deletion of *prrt2* reduces the amount of glutamate release, but not GABA release, in a gene copy-number-dependent manner. Neurotransmitter levels of Prrt2 WT (black bars), Het (grey bars), and KO (white bars) of P5 cerebellar neurons measured by ELISA. **a** Glutamate release: Cerebellar granule cells for Prrt2 WT *n* = 36, Het (*p = 0.0105, *n* = 32) and KO (****p < 0.0001), *n* = 45. **b** GABA release: Cerebellar granule cells for Prrt2 WT *n* = 8, Het (*p* = NS, *n* = 5) and KO (*p* = NS, *n* = 6). Results were adjusted for protein levels and expressed as a percentage of total neurotransmitter level (neurotransmitter release/neurotransmitter release + lysate level × 100). One-way ANOVA, Tukey’s multiple comparisons to Prrt2 WT neurons

**Fig. 4:**
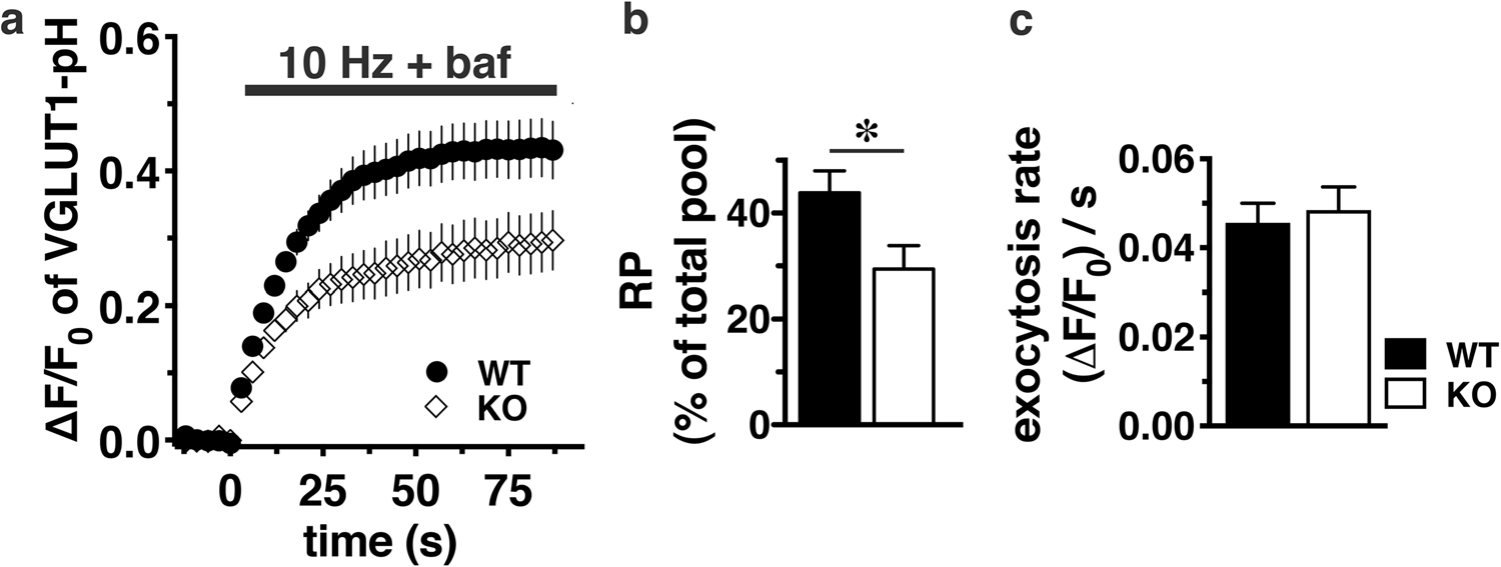
Deletion of *prrt2* reduces the amount of VGLUT1-pH in the RP, but not the rate of exocytosis. **a** The time course of VGLUT1-pH fluorescence changes in response to electrical stimulation at 10 Hz 90 s (bar) in the presence of 5 µM bafilomycin (baf) in transfected hippocampal synaptic boutons from WT (black circles) or *prrt2* KO mice (white triangles), normalized to the total fluorescence in each bouton in the presence of 50 mM NH_4_Cl applied after stimulation. The fluorescence of VGLUT1-pH increases rapidly upon stimulation and reaches a plateau which represents the fraction of VGLUT1-pH in RP. **b** Loss of *prrt2* decreases the amount of VGLUT1-pH in the RP (KO, white bars 0.2940 ± 0.0442), compared to that in WT (black bars, 0.438 ± 0.0419, *p < 0.05). **c** The rate of VGLUT1-pH exocytosis is not significantly altered by loss of *prrt2* [(Δ*F*/*F*_0_)/s: WT 0.0459 ± 0.0044; and KO 0.0485 ± 0.0052, *p* > 0.05)]. Data are means ± SEM of Δ*F*/*F*_0_ normalized to total fluorescence. Data are from 27 to 91 boutons per coverslips from 7 to 10 coverslips from three independent cultures

The reductions in expression of synaptic proteins in the Prrt2 KO and Pkd mice seen at P5 correlate with a dose-dependent reduction in glutamate release from cerebellar granule cells harvested from P5 mice (Fig. 3). By contrast, there is no change in the low level of GABA release in the Pkd or Prrt2 KO mice from the small number GABAergic neurons (likely Purkinje cells or interneurons) that remain in the culture of cerebellar granule cells. In a fluorescence-based assay of synaptic vesicle release (Fig. 4), using a pH-sensitive tagged vGLUT1, rates of vesicle release are similar between Prrt2 KO and wild-type mice, but the extent of vesicle release is greatly reduced in the absence of Prrt2 (*p* < 0.05).

**Fig. 5:**
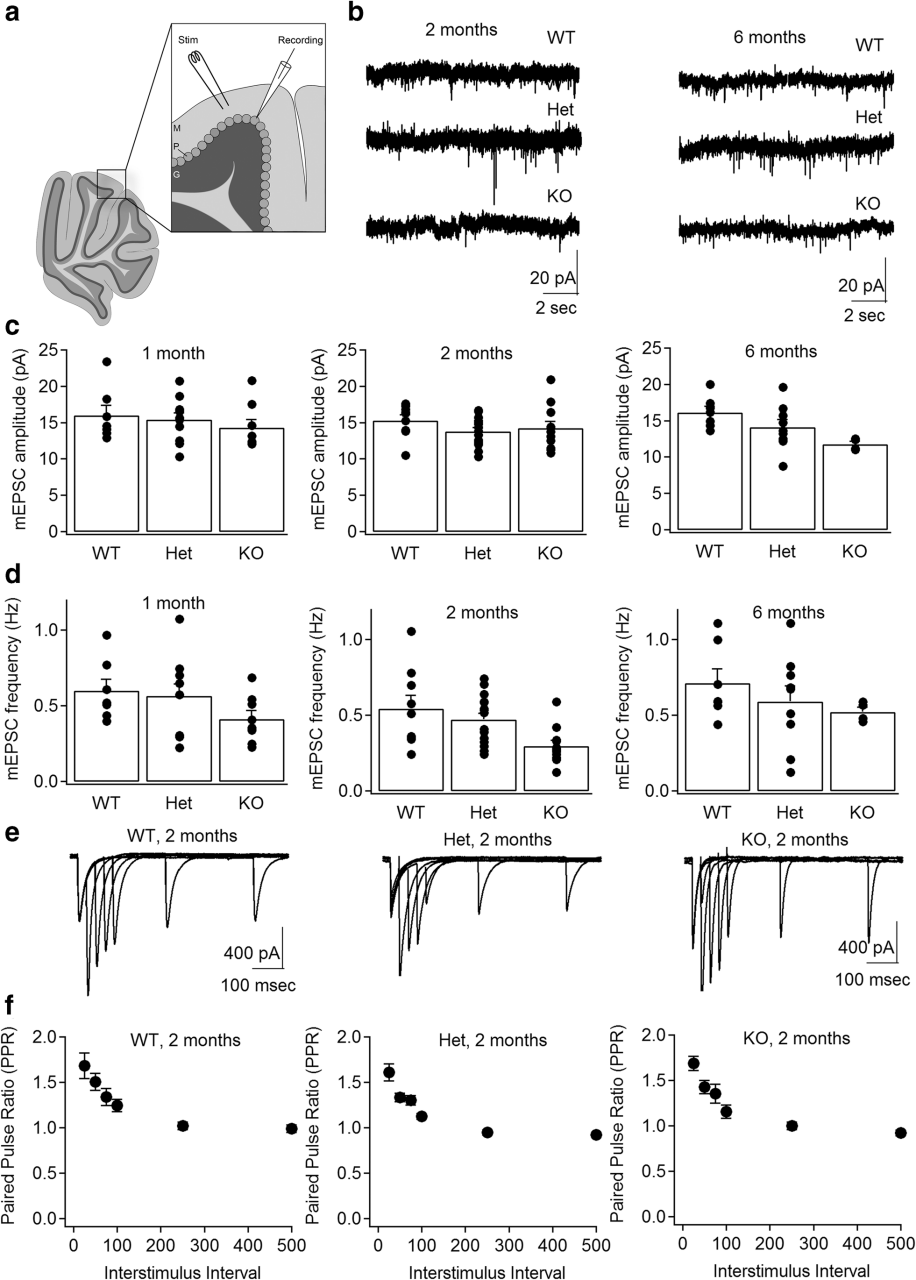
Cerebellar synaptic transmission altered at 2 months in PRRT2 mice. Cerebellar Purkinje cells were recorded in the whole-cell, voltage-clamp mode in slices from PRRT WT, Het, and KO mice at ages 1, 2, and 6 months. A stimulating electrode was placed in the nearby molecular layer. **a** Schematic of the recording configuration. **b**–**d** Miniature excitatory postsynaptic currents (mEPSCs) recorded in the presence of picrotoxin and tetrodotoxin. **b** Traces of mEPSCs in representative cells from WT, Het, and KO mice at 2 and 6 months. **c**, **d** Average mEPSC amplitudes **c** and frequencies (**d**) measured at 1 month (WT: *N* = 4/*n* = 7; Het: *N* = 7/*n* = 11; KO: *N* = 3/*n* = 8), 2 months (WT: *N* = 5/*n* = 10; Het: *N* = 9/*n* = 15; KO: *N* = 6/*n* = 11) and 6 months (WT: *N* = 4/*n* = 8; Het: *N* = 5/*n* = 9; KO: *N* = 1/*n* = 4). Individual neurons are represented by the overlaid dots. **e**–**f** Electrically evoked excitatory postsynaptic currents, measured in the presence of picrotoxin at 2 months. **e** Pairs of overlaid EPSCs evoked at different interstimulus intervals (ISIs) in representative cells from WT, Het, and KO slices. **f** Average paired-pulse ratio (PPR) at different ISIs in WT (*N* = 3/*n* = 9), Het (*N* = 6/*n* = 20), and KO slices (*N* = 5/*n* = 12). *N* refers to animals, *n* to cells. Data represented as average + / − SEM

Given the age-dependent alterations in presynaptic protein expression and in vitro glutamate release, we next examined whether synaptic transmission was altered in the Prrt2 mouse model. Several lines of evidence suggest the cerebellum as a likely locus for alterations in synaptic function, including strong PRRT2 expression, age-dependent changes in expression, and linkage to dystonic movements. [14–16] To assay synaptic transmission in the cerebellum of Pkd and KO mice, we performed whole-cell voltage-clamp electrophysiological recordings from cerebellar Purkinje cells in ex vivo slices of the cerebellum (Fig. 5a), again focusing on age-dependent changes. We first measured miniature excitatory postsynaptic currents (mEPSCs) onto Purkinje cells (Fig. 5b), comparing wild-type, heterozygous, and knockout mice at 1, 2, and 6 months. Typically, the amplitude of mEPSCs reflects postsynaptic function, whereas mEPSC frequency reflects presynaptic function, including the number of inputs and/or their probability of release. The amplitude of mEPSCs does not significantly differ between the three Prrt2 genotypes at any of the three ages (Fig. 5c; *p* = 0.42; *p* = 0.23, *p* = 0.68; Kruskal–Wallis test). However, at 2 months (but not 6 months), the frequency of mEPSCs is reduced in slices from heterozygous and knockout mice, compared to those from wild-type mice (Fig. 5d; *p* = 0.0096, Kruskal–Wallis test). These results suggest that loss of Prrt2 yields reduced presynaptic glutamatergic input onto Purkinje cells at 2 months of age, which resolves by 6 months of age.

Age-dependent changes in glutamatergic synapses in Prrt2 mice suggest the possibility that the loss of Prrt2 disrupts or delays normal synaptic development, resulting in either a reduction in functional synaptic contacts or alterations in their individual function. To determine whether glutamatergic synapses onto Purkinje cells have altered release properties, we next measured evoked synaptic release, focusing on the 2-month time point. We activated inputs by local electrical stimulation through a glass stimulating electrode placed in the molecular layer (Fig. 5e), focusing on a common indicator of presynaptic release probability, the paired-pulse ratio (PPR). At low release probability synapses, two pulses in close succession produce paired-pulse facilitation, whereas at high release probability synapses, two pulses in close succession produce paired-pulse depression. In wild-type slices, we gave two pulses at a variety of interstimulus intervals (Fig. 5e), and measured PPR. At short interstimulus intervals, we find a strong paired-pulse facilitation, whereas there is little if any facilitation at intervals > 150 ms (Fig. 5f). We then compared the PPR curves of Purkinje cells recorded in wild-type, heterozygous, and knockout Prrt2 mice. We found the PPR curves are not significantly different among the three genotypes (Fig. 5f). Taken together, these findings suggest that, though there is an age-dependent reduction in synaptic input onto Purkinje cells in Prrt2 mice, this is not likely to be driven by a global reduction in the probability of release.

**Fig. 6:**
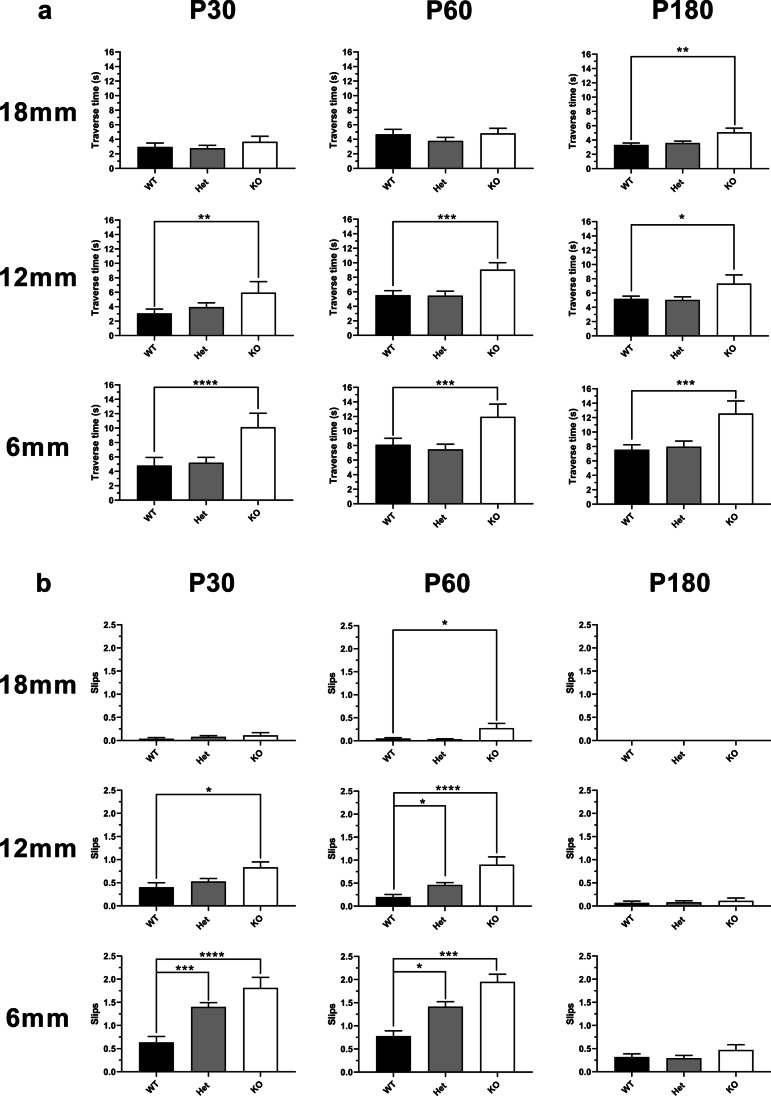
Balance and motor coordination assessed on the balance beam. Transient loss of coordination, as measured by increased slipping on the balance beam test, is apparent at P30, and seems to persist and even worsen at P60, but disappears by P180. Prolonged balance beam traverse time becomes more apparent at later time points (P60 and P180). Graphs represent traverse time (s) and slips. Bars represent the mean + / − SEM of the final days (5–8 trials), post training on the beam. (**a**) Traverse time and (**b**) slips: P30: WT (black bar) *n* = 13, Het (grey bar) *n* = 28, and KO (white bar) *n* = 9. P60: WT (black bar) *n* = 27, Het (grey bar) *n* = 34, and KO (white bar) *n* = 10. P180: WT (black bar) *n* = 15, Het (grey bar) *n* = 22, and KO (white bar) *n* = 9. The maximum traverse time was 20 s. Traverse time greater than 20 s the mouse fails and was counted as 20 s. One-way ANOVA, Tukey’s multiple comparisons, compared to WT. (*p < 0.05, **p < 0.01, ***p < 0.001, ****p < 0.0001)

We then proceeded to assess the motor phenotypes of wild-type, Pkd, and Prrt2 KO mice at different ages. All three genotypes were challenged with the learned task of crossing balance beams of progressively narrower widths, at ages P30, P60, and P180 (Fig. 6a–c). With P30 mice, there is an increase in traverse time as the beam is narrowed in width from 18 to 12 mm and 6 mm, though this difference is only seen in the Prrt2 KO mice. The Prrt2 KO mice also have increased slips compared to wild-type on the 12-mm and 6-mm beams, while the Pkd mice show an increased frequency of slips only on the narrowest beam. At P60, the longer traverse time in the Prrt2 KO versus the wild-type and Pkd mice is maintained, while the differences in slipping become more pronounced. In particular, an increased rate of slips is apparent in the Prrt2 KO mice even on the widest beam of 18-mm diameter. When crossing the narrower 12-mm and 6-mm beams, Pkd and Prrt2 KO mice have more slips than the wild-type mice in a PRRT2 dose-dependent pattern. Finally, at P180, the slower traverse time for Prrt2 KO mice is still detectable, and present even across the 18-mm beam, while there is no longer a significant difference in the rate of slips on any of the beams between wild-type, Pkd, and Prrt2 KO mice. Thus, the slower traverse time of the knockout mice persists across the range of ages tested, while the rate of slips is increased at P30, peaks at P60, and disappears by P180.

**Fig. 7:**
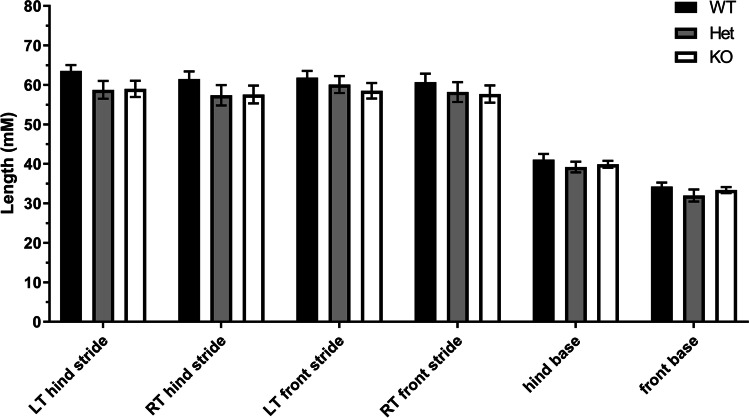
Gait and stride analysis of mice from all three genotypes revealed no significant differences in stride length of forelimbs or hindlimbs in Pkd mice compared to those in wild-type at P60. WT (black bar) *n* = 11, Het (grey bar) *n* = 10, and KO (white bar) *n* = 7. No significant differences by 2-way ANOVA

Gait analysis of mice from all three genotypes reveals no significant differences in stride length of forelimbs or hindlimbs in Pkd mice compared to those in wild-type (Fig. 7). Similarly, there is no difference in rotarod performance at p30 and p80 between the wild-type and Pkd mice (data not shown).

## Discussion

The age dependence of paroxysmal symptoms in PRRT2-related diseases is a widely reported but poorly understood phenomenon. Efforts of several groups have helped to elucidate the mechanisms of PRRT2 mutations in synaptic function, through a combination of knockdown [8, 9, 28] and knockout [5, 9, 14–16] approaches in rodent models, along with immunohistochemistry, electrophysiology, and behavioral experiments. These studies have shown a pattern of PRRT2 expression restricted to the brain and spinal cord, and most prominent in the cerebellum and cortex. Our model affirms the distribution of *Prrt2* mRNA and Prrt2 protein throughout the central nervous system with an allelic mutation–dependent reduction in Pkd and Prrt2 KO mice (Fig. 1). This reduction in PRRT2 correlates with reduction in synaptic proteins. The level of some synaptic proteins decreased in a dose-dependent manner, while others were maximally reduced with Prrt2 haploinsufficency. These biochemical changes are most pronounced at P30 and P60, the same age window where presynaptic glutamatergic input is reduced. At the ultrastructural level, PRRT2 is firmly established as a presynaptic protein, [8, 26, 29] though recent work argues for an important role in modulation of voltage-gated sodium channels at the axon initial segment. [11] At the synapse, PRRT2 co-localizes with SNAP-25 and syntaxin-1, [10] and loss of PRRT2 leads to a decrease in synaptic vesicle density. [8, 30].

Our finding of reduced levels of multiple synaptic proteins in the Pkd and Prrt2 KO mice, compared to that in the wild-type, could suggest a globally reduced number of neurons or reduced synaptic density. However, the unchanged levels of some synaptic proteins, such as PNKD, among the three genotypes, as well as the similarity to wild-type levels in older (P60 and P180) mice would argue against a reduced number of neurons. Furthermore, our electrophysiological data support decreased synaptic input at earlier time points, without an effect on release probability, findings also consistent with reduced synaptic density. Rather, our results are consistent with those of Liu et al. [26] and Valente et al. [29] who found that PRRT2 knockdown decreased synaptic density after birth. Indeed, Liu et al. noted delays in neuronal migration, but that neurons eventually reach their target, a model that would also be compatible with our findings. Thus, our western blot data would support Liu et al.’s suggestion that loss of Prrt2 slows synaptic development, with a reduced number of synapses at early time points. Our western blotting data cannot distinguish whether deficiency of Prrt2 leads to transcriptional or translational repression of other synaptic proteins, or to changes in protein turnover.

There has been some variability in findings among rodent models of PRRT2-related diseases, with most publications showing primarily synaptic localization, and one recent publication showing a role for PRRT2 in regulation of voltage-gated sodium channels in the axon. [11] In addition, while there is consensus that PRRT2 modulates glutamate release at excitatory synapses, some groups have identified changes in inhibitory neurotransmitter release. [10, 29] It should be noted that altered inhibitory neuron physiology in Prrt2 KO mice was identified in hippocampal, not cerebellar neurons, which likely accounts for differences between our findings and those reports. Our data are consistent with studies focused on the cerebellum in Prrt2 KO and Pkd mice, [15] in spite of differences in technical approaches, while hippocampal electrophysiology suggests a more complex increase in cortical excitability [29] due to altered activity at both excitatory and inhibitory synapses. Dyskinesia in PKD seems to be mechanistically distinct from paroxysmal non-kinesigenic dyskinesia (PNKD), which is caused by dominant mutations in MR-1, and is associated with alterations in dopaminergic signaling in the striatum. [31] It remains an open question whether there are convergent metabolic and anatomic pathways shared by the various dyskinesias.

Increased slipping on the balance beam test, which may be due to transient balance and coordination deficits, including dyskinesias, is apparent at P30, and seems to persist and even worsen at P60, but disappears by P180. Prolonged balance beam traverse time, a more complex measure that may reflect sustained deficits in coordination, balance, anxiety, and motivation, becomes more apparent at later time points (P60 and P180). Thus, the reduced levels of synaptic proteins at early time points do not correlate with impaired coordination. Our findings of more frequent slips and longer traverse time on progressively narrower balance beams are similar to the findings of Tan et al., [15] though they studied mice only at 6 weeks of age. Michetti et al., [14] on the other hand, examined motor coordination at very early time points, from P4 to P16, and found that loss of balance peaked at P8, while backwalking and grooming increased during the first 2 weeks of life. The reduced expression levels of synaptic proteins at earlier time points in the Pkd and Prrt2 KO mice may reflect a developmental abnormality of the synapse, which is independent of the transient uncoordination/dyskinetia phenotype that appears early and persists into adulthood. Our data suggest that this transient uncoordination/dyskinetia may peak, while difficulties with balance beam traversal, reflecting a more sustained impairment of coordination, persist in the Prrt2 KO mice, consistent with the more severe phenotype seen in the rare patients with biallelic mutations in PRRT2. [7]

Thus, there appear to be three phenotypic windows: early delayed synaptic development, which may be associated with increased seizure propensity; a peak in transient uncoordination/dyskinesia around P60 that attenuates by P180; and a persistent uncoordination phenotype that is present at P180 and beyond in Prrt2 KO mice. Delayed cortical migration is associated with many severe epileptic encephalopathies, and may explain why there is a developmental window for epilepsy in individuals with PRRT2 mutations. More recent data from Fruscione et al. [11] implicate a sodium channel inhibitory role for PRRT2, which becomes less inhibited in disease-associated PRRT2 mutations, and which offers an explanation for the sensitivity of both seizures and dyskinesias to sodium channel–blocking agents like carbamazepine. It remains to be further clarified, however, why this effect of PRRT2 manifests as seizures in infancy and kinesigenic dyskinesia in later childhood and adulthood. Perhaps further work may elucidate the infantile vulnerability of cortex compared to the later vulnerability of cerebellum, which appears to be the source of dyskinesias. [15].

## Data Availability

Data and mouse model will be made available upon request.

## References

[1] Wang JL, Cao L, Li XH, Hu ZM, Li JD, Zhang JG, Liang Y, San A, Li N, Chen SQ, Guo JF, Jiang H, Shen L, Zheng L, Mao X, Yan WQ, Zhou Y, Shi YT, Ai SX, Dai MZ, Zhang P, Xia K, Chen SD, Tang BS (2021). Identification of PRRT2 as the causative gene of paroxysmal kinesigenic dyskinesias. Brain.

[2] Chen WJ, Lin Y, Xiong ZQ, Wei W, Ni W, Tan GH, Guo SL, He J, Chen YF, Zhang QJ, Li HF, Lin Y, Murong SX, Xu J, Wang N, Wu ZY (2011). Exome sequencing identifies truncating mutations in PRRT2 that cause paroxysmal kinesigenic dyskinesia. Nat Genet.

[3] van Vliet R, Breedveld G, de Rijk-van AJ, Brilstra E, Verbeek N, Verschuuren-Bemelmans C, Boon M, Samijn J, Diderich K, van de Laar I, Oostra B, Bonifati V, Maat-Kievit A (2012). PRRT2 phenotypes and penetrance of paroxysmal kinesigenic dyskinesia and infantile convulsions. Neurology.

[4] Cloarec R, Bruneau N, Rudolf G, Massacrier A, Salmi M, Bataillard M, Boulay C, Caraballo R, Fejerman N, Genton P, Hirsch E, Hunter A, Lesca G, Motte J, Roubertie A, Sanlaville D, Wong SW, Fu YH, Rochette J, Ptácek LJ, Szepetowski P (2012). PRRT2 links infantile convulsions and paroxysmal dyskinesia with migraine. Neurology.

[5] Lee HY, Huang Y, Bruneau N, Roll P, Roberson ED, Hermann M, Quinn E, Maas J, Edwards R, Ashizawa T, Baykan B, Bhatia K, Bressman S, Bruno MK, Brunt ER, Caraballo R, Echenne R, Fejerman N, Frucht S, Gurnett CA, Hirsch E, Houlden H, Jankovic J, Lee WL, Lynch DR, Mohammed S, Müller U, Nespeca MP, Renner D, Rochette J, Rudolf G, Saiki S, Soong BW, Swoboda KJ, Tucker S, Wood N, Hanna M, Bowcock AM, Szepetowski P, Fu YH, Ptáček LJ (2012). Mutations in the gene PRRT2 cause paroxysmal kinesigenic dyskinesia with infantile convulsions. Cell Rep.

[6] Bruno MK, Hallett M, Gwinn-Hardy K, Sorensen B, Considine E, Tucker S, Lynch DR, Mathews KD, Swoboda KJ, Harris J, Soong BW, Ashizawa T, Jankovic J, Renner D, Fu YH, Ptacek LJ (2004). Clinical evaluation of idiopathic paroxysmal kinesigenic dyskinesia: new diagnostic criteria. Neurology.

[7] Gardiner AR, Bhatia KP, Stamelou M, Dale RC, Kurian MA, Schneider SA, Wali GM, Counihan T, Schapira AH, Spacey SD, Valente EM, Silveira-Moriyama L, Teive HA, Raskin S, Sander JW, Lees A, Warner T, Kullmann DM, Wood NW, Hanna M, Houlden H (2012). PRRT2 gene mutations: from paroxysmal dyskinesia to episodic ataxia and hemiplegic migraine. Neurology.

[8] Dale RC, Gardiner A, Antony J, Houlden H (2012). Familial PRRT2 mutation with heterogeneous paroxysmal disorders including paroxysmal torticollis and hemiplegic migraine. Dev Med Child Neurol.

[9] Delcourt M, Riant F, Mancini J, Milh M, Navarro V, Roze E, Humbertclaude V, Korff C, Des Portes V, Szepetowski P, Doummar D, Echenne B, Quintin S, Leboucq N, Singh Amrathlal R, Rochette J, Roubertie A (2015). Severe phenotypic spectrum of biallelic mutations in PRRT2 gene. J Neurol Neurosurg Psychiatry.

[10] Valente P, Castroflorio E, Rossi P, Fadda M, Sterlini B, Cervigni RI, Prestigio C, Giovedì S, Onofri F, Mura E, Guarnieri FC, Marte A, Orlando M, Zara F, Fassio A, Valtorta F, Baldelli P, Corradi A, Benfenati F (2016). PRRT2 is a key component of the Ca(2+)-dependent neurotransmitter release machinery. Cell Rep.

[11] Li M, Niu F, Zhu X, Wu X, Shen N, Peng X, Liu Y (2015). PRRT2 mutant leads to dysfunction of glutamate signaling. Int J Mol Sci.

[12] Mo J, Wang B, Zhu X, Wu X, Liu Y (2019). PRRT2 deficiency induces paroxysmal kinesigenic dyskinesia by influencing synaptic function in the primary motor cortex of rats. Neurobiol Dis.

[13] Fruscione F, Valente P, Sterlini B, Romei A, Baldassari S, Fadda M, Prestigio C, Giansante G, Sartorelli J, Rossi P, Rubio A, Gambardella A, Nieus T, Broccoli V, Fassio A, Baldelli P, Corradi A, Zara F, Benfenati F (2018). PRRT2 controls neuronal excitability by negatively modulating Na+ channel 1.2/1.6 activity. Brain.

[14] Schubert J, Paravidino R, Becker F, Berger A, Bebek N, Bianchi A, Brockmann K, Capovilla G, Dalla Bernardina B, Fukuyama Y, Hoffmann GF, Jurkat-Rott K, Anttonen AK, Kurlemann G, Lehesjoki AE, Lehmann-Horn F, Mastrangelo M, Mause U, Müller S, Neubauer B, Püst B, Rating D, Robbiano A, Ruf S, Schroeder C, Seidel A, Specchio N, Stephani U, Striano P, Teichler J, Turkdogan D, Vigevano F, Viri M, Bauer P, Zara F, Lerche H, Weber YG (2012). PRRT2 mutations are the major cause of benign familial infantile seizures. Hum Mutat.

[15] Ebrahimi-Fakhari D, Saffari A, Westenberger A, Klein C (2015). The evolving spectrum of *PRRT2*-associated paroxysmal diseases. Brain.

[16] Michetti C, Castroflorio E, Marchionni I, Forte N, Sterlini B, Binda F, Fruscione F, Baldelli P, Valtorta F, Zara F, Corradi A, Benfenati F (2017). The PRRT2 knockout mouse recapitulates the neurological diseases associated with PRRT2 mutations. Neurobiol Dis.

[17] Tan GH, Liu YY, Wang L, Li K, Zhang ZQ, Li HF, Yang ZF, Li Y, Li D, Wu MY, Yu CL, Long JJ, Chen RC, Li LX, Yin LP, Liu JW, Cheng XW, Shen Q, Shu YS, Sakimura K, Liao LJ, Wu ZY, Xiong ZQ (2018). PRRT2 deficiency induces paroxysmal kinesigenic dyskinesia by regulating synaptic transmission in cerebellum. Cell Res.

[18] Calame DJ, Xiao J, Khan MM, Hollingsworth TJ, Xue Y, Person AL, LeDoux MS (2020). Presynaptic PRRT2 deficiency causes cerebellar dysfunction and paroxysmal kinesigenic dyskinesia. Neuroscience.

[19] Capoccia S, Maccarinelli F, Buffoli B, Rodella LF, Cremona O, Arosio P, Cirulli F (2015). Behavioral characterization of mouse models of neuroferritinopathy. PLoS ONE.

[20] K Kato PS Puttfarcken WE Lyons JT Coyle 1991Developmental time course and ionic dependence of kainate-mediated toxicity in rat cerebellar granule cell culturesJ Pharmacol Exp Ther 256 1 402 411 1846423

[21] Griffiths R, Malcolm C, Ritchie L, Frandsen A, Schousboe A, Scott M, Rumsby P, Meredith C (1997). Association of c-fos mRNA expression and excitotoxicity in primary cultures of mouse neocortical and cerebellar neurons. J Neurosci Res.

[22] Vale C, Pomés A, Rodríguez-Farré E, Suñol C (1997). Allosteric interactions between gamma-aminobutyric acid, benzodiazepine and picrotoxinin binding sites in primary cultures of cerebellar granule cells. Differential effects induced by gamma- and delta-hexachlorocyclohexane. Eur J Pharmacol.

[23] Vale C, Fonfría E, Bujons J, Messeguer A, Rodríguez-Farré E, Suñol C (2003). The organochlorine pesticides gamma-hexachlorocyclohexane (lindane), alpha-endosulfan and dieldrin differentially interact with GABA(A) and glycine-gated chloride channels in primary cultures of cerebellar granule cells. Neuroscience.

[24] Voglmaier SM, Kam K, Yang H, Fortin DL, Hua Z, Nicoll RA, Edwards RH (2006). Distinct endocytic pathways control the rate and extent of synaptic vesicle protein recycling. Neuron.

[25] Gandhi SP, Stevens CF (2003). Three modes of synaptic vesicular recycling revealed by single-vesicle imaging. Nature.

[26] Li H, Foss SM, Dobryy YL, Park CK, Hires SA, Shaner NC, Tsien RY, Osborne LC, Voglmaier SM (2011). Concurrent imaging of synaptic vesicle recycling and calcium dynamics. Front Mol Neurosci.

[27] Hua Z, Leal-Ortiz S, Foss SM, Waites CL, Garner CC, Voglmaier SM, Edwards RH (2011). v-SNARE composition distinguishes synaptic vesicle pools. Neuron.

[28] Liu YT, Nian FS, Chou WJ, Tai CY, Kwan SY, Chen C, Kuo PW, Lin PH, Chen CY, Huang CW, Lee YC, Soong BW, Tsai JW (2016). PRRT2 mutations lead to neuronal dysfunction and neurodevelopmental defects. Oncotarget.

[29] Valente P, Romei A, Fadda M, Sterlini B, Lonardoni D, Forte N, Fruscione F, Castroflorio E, Michetti C, Giansante G, Valtorta F, Tsai JW, Zara F, Nieus T, Corradi A, Fassio A, Baldelli P, Benfenati F (2019). Constitutive inactivation of the PRRT2 gene alters short-term synaptic plasticity and promotes network hyperexcitability in hippocampal neurons. Cereb Cortex.

[30] Lee HY, Nakayama J, Xu Y, Fan X, Karouani M, Shen Y, Pothos EN, Hess EJ, Fu YH, Edwards RH, Ptácek LJ (2012). Dopamine dysregulation in a mouse model of paroxysmal nonkinesigenic dyskinesia. J Clin Invest.

